# Identification of cross reactive T cell responses in adenovirus based COVID 19 vaccines

**DOI:** 10.1038/s41541-024-00895-z

**Published:** 2024-06-05

**Authors:** Joshua Gardner, Simon Timothy Abrams, Cheng-Hock Toh, Alan L. Parker, Charlotte Lovatt, Phillip L. R. Nicolson, Steve P. Watson, Sophie Grice, Luisa Hering, Munir Pirmohamed, Dean J. Naisbitt

**Affiliations:** 1 Centre for Drug Safety Science, Department of Pharmacology and Therapeutics, University of Liverpool, Liverpool, United Kingdom; 2 Institute of Infection, Veterinary Sciences and Ecological Sciences, University of Liverpool, Liverpool, United Kingdom; 3 Division of Cancer and Genetics, School of Medicine, Cardiff University, Cardiff, United Kingdom; 4 Institute of Cardiovascular Sciences, University of Birmingham, Birmingham, United Kingdom; 5 Department of Haematology, Queen Elizabeth Hospital, Birmingham, United Kingdom

**Keywords:** Vaccines, Immunological memory, Lymphocyte activation, Drug safety

## Abstract

Vaccination has proven to be a valuable tool to combat SARS-CoV-2. However, reports of rare adverse reactions such as thrombosis/thrombocytopenia syndrome after ChAdOx1 nCoV-19 vaccination have caused scientific, public and media concern. ChAdOx1 was vectorised from the Y25 chimpanzee adenovirus, which was selected due to low human seroprevalence to circumvent pre-existing immunity. In this study, we aimed to explore patterns of T-cell activation after SARS-CoV-2 COVID-19 vaccine exposure in vitro using PBMCs collected from pre-pandemic ChAdOx1 nCoV-19 naïve healthy donors (HDs), and ChAdOx1 nCoV-19 and Pfizer vaccinated controls. PBMCs were assessed for T-cell proliferation using the lymphocyte transformation test (LTT) following exposure to SARS-CoV-2 COVID-19 vaccines. Cytokine analysis was performed via intracellular cytokine staining, ELISpot assay and LEGENDplex immunoassays. T-cell assays performed in pre-pandemic vaccine naïve HDs, revealed widespread lymphocyte stimulation after exposure to ChAdOx1 nCoV-19 (95%), ChAdOx-spike (90%) and the Ad26.COV2. S vaccine, but not on exposure to the BNT162b2 vaccine. ICS analysis demonstrated that CD4^+^ CD45RO^+^ memory T-cells are activated by ChAdOx1 nCoV-19 in vaccine naïve HDs. Cytometric immunoassays showed ChAdOx1 nCoV-19 exposure was associated with the release of proinflammatory and cytotoxic molecules, such as IFN-γ, IL-6, perforin, granzyme B and FasL. These studies demonstrate a ubiquitous T-cell response to ChAdOx1 nCoV-19 and Ad26.COV2. S in HDs recruited prior to the SARS-CoV-2 pandemic, with T-cell stimulation also identified in vaccinated controls. This may be due to underlying T-cell cross-reactivity with prevalent human adenoviruses and further study will be needed to identify T-cell epitopes involved.

## Introduction

The development of effective vaccines against Severe Acute Respiratory Syndrome Coronavirus 2 (SARS-CoV-2) was crucial in tackling the Covid-19 pandemic. Several vaccines were developed with strong efficacy for protection against SARS-CoV-2 using both existing and novel vaccine technologies^1–3^. Licensed viral vector vaccines include ChAdOx1 nCoV-19 (AZD1222) and Ad26.COV2. S (Janssen/J&J) which are replication deficient and are based on a modified-chimpanzee adenovirus (Y25) and human adenovirus type 26 (Ad26), respectively^4,5^. By contrast, other vaccines, such as the BNT162b2 (BioNTech/Pfizer) vaccine, followed more novel vaccine technology involving the use of lipid encapsulated messenger RNA (mRNA) encoding SARS-CoV-2 spike proteins^6^. Collectively, data from large scale clinical trials have documented strong immunogenicity in the form of robust antibody and T-cell responses (CD4+ and CD8+) following vaccination and subsequent exposure to SARS-CoV-2 spike proteins^5,7,8^. However, data describing the intrinsic immunogenicity of Covid-19 vaccines is currently limited and little is known about pre-existing levels of cellular immunity to adenoviral vector platforms, such as ChAdOx1.

The adenoviral vector vaccines, ChAdOx1 nCoV-19 and Ad26.COV2. S, have been associated with the development of thrombosis and thrombocytopenia syndrome (TTS). TTS is also known as vaccine-induced immune thrombotic thrombocytopenia (VITT) when a vaccine has been implicated. Studies have indicated that adenoviral vector vaccination is responsible for approximately 98.5% of all VITT cases^9^, with ChAdOx1 nCoV-19 vaccination linked with a higher incidence (1/100,000) when compared to Ad26.COV2. S vaccination (1/500,000)^10–12^. Although there have been reports of VITT associated with the use of the mRNA vaccines, these are rare, and may represent background events, and thus causality is unclear^13–15^.

In addition to thrombotic events, immediate hypersensitivity reactions following SARS-CoV-2 vaccination have also been reported for the ChAdOx1 nCoV-19, Ad26.COV2. S and BNT162b2 vaccines^16,17^, with allergy to vaccine excipients cited as common cause of anaphylaxis^18^. Furthermore, cases of delayed onset hypersensitivity reactions to SARS-CoV-2 vaccines, including drug reaction with eosinophilia and systemic symptoms (DRESS) in severe cases^19,20^ and delayed injection site reactions^21^ have been identified, but again causality is unclear in many cases^22^.

Prior exposure to human adenoviruses is well established^23^. Initial development of adenoviral vectors focused almost entirely on human adenovirus type 5 (Ad5), but the high prevalence of prior exposure (60–98%) can increase neutralising antibody titres and reduce efficacy^24,25^. The recombinant viral-vector based vaccines, ChAdOx1 nCoV-19 and Ad26.COV2. S, are produced using human cell lines and were selected due to the low population seroprevalence to circumvent pre-existing immunity. ChAdOx1 encompasses a chimpanzee adenoviral isolate (Y25) and its suitability for use as a vaccine candidate has been studied over several decades with evidence of low seroprevalence in humans^4,26^. Similarly, Ad26.COV2. S is associated with significantly lower seroprevalence in the general population than other candidate human adenoviral vectors^27^.

The aim of the present study was to investigate the frequency and phenotype of T-cell responses to ChAdOx1 and other SARS-CoV-2 COVID-19 vaccines using peripheral blood mononuclear cells (PBMC) stimulation assays. This allows for the detection of proliferative and cytokine-based memory T-cell responses and any underlying T-cell cross-reactivity^28^. Proliferation and cytokine analysis were conducted using both pre- and post-pandemic PBMC samples, encompassing (i) healthy, vaccine naïve donors, (ii) ChAdOx1 nCoV-19 vaccinated controls and (iii) ChAdOx1 nCoV-19 naïve, Pfizer vaccinated controls.

## Results

### Memory T-cell responses to ChAdOx1 nCoV-19 and Ad26 are present in pre-pandemic vaccine naive healthy donor PBMC

**Fig. 1:**
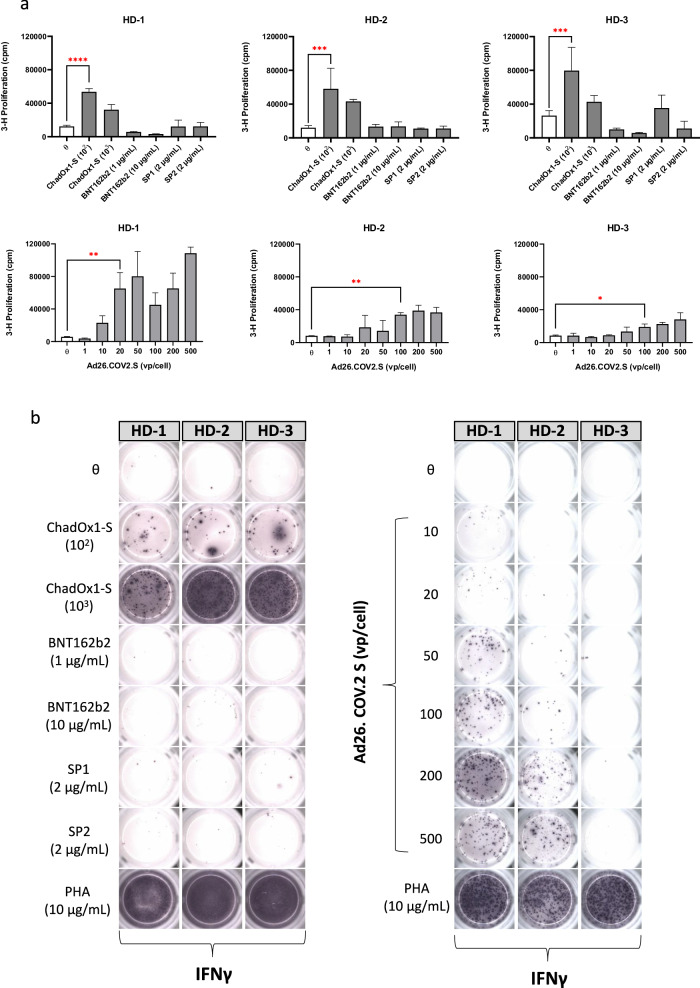
T-cell responses to ChAdOx1 nCoV-19, Ad26.COV2. S, BNT162b2 and SARS-CoV-2 spike protein peptide pools in healthy, vaccine naïve donors determined by lymphocyte transformation test. **a** Proliferative T-cell responses to ChAdOx1 nCoV-19 (vp/cell), BNT162b2 (µg/mL), Ad26.COV2. S (vp/cell) SP1 and SP2 peptide pools (µg/mL) after 5-day exposure (*n* = 3). T-cell proliferation was is given by counts per minute (cpm) and determined by 16 h pulsing with tritiated [^3^H]-thymidine (0.5 µCi/well) before scintillation counting. Statistical significance was determined using one-way ANOVA (*p < 0.01, **p < 0.001 ***p < 0.001, ****p < 0.0001). Data are presented as mean ± standard deviation of 3 experimental replicates. **b** Cytokine release (IFN-γ) after 48 h exposure to ChAdOx1 nCoV-19 (100–1000 vp/cell), BNT162b2 (1–10 µg/mL), SARS-CoV-2 spike protein peptide pools (SP1; 2 µg/mL and SP2; 2 µg/mL) and the Ad26.COV2. S vaccine (0–500 vp/cell) in pre-pandemic vaccine naïve HDs (*n* = 3) assessed using the ELISpot assay.

To investigate frequency and phenotype of memory T-cell responses to a range of SARS-CoV-2 COVID-19 vaccines a panel of vaccine naïve healthy donors (HDs) were randomly selected from a cohort of cryopreserved PBMC collected by the Liverpool Healthy Donor Biobank in 2013 (Fig. 1). In the panel of 3 HDs initially studied, high levels of lymphocyte proliferation were identified in each HD after 5-day exposure to ChAdOx1 nCoV-19 and Ad26.COV2. S using the lymphocyte transformation test (Fig. 1a). For ChAdOx1 nCoV-19, maximal T-cell stimulation (cpm > 4 × 10^4^) was present at 10^3^ vp/cell, whereas Ad26.COV2. S produced maximal stimulation at 500 vp/cell (cpm > 3 × 10^4^). Memory T-cell responses using the LTT were absent after exposure to BNT162b2 (1–10 µg/mL). No evidence of T-cell activation was found after PBMC incubation with SARS-CoV-2 spike protein peptide pools (SP1 and SP2), providing confirmation that HDs selected had not previously been exposed to SARS-CoV-2. Furthermore, cytokine release was observed after 48 h stimulation with ChAdOx1 nCoV-19 and Ad26.COV2. S, but not after exposure to BNT162b2, SP1 and SP2 (Fig. 1b). Differences in T-cell reactivity following exposure to Ad26.COV2. S were observed in HD-3, with cytokine release notably absent. This may be a result of the weak proliferative responses associated with HD-3 and general inter-individual variability between healthy donor samples with differing levels of prior exposure. After removal of the T-cell component (CD3^+^ T-cells) from whole PBMC populations, no lymphocyte proliferation or cytokine release (IFN-γ) was observed after stimulation with ChAdOx1 nCoV-19 (Supplementary Fig. 1).

### Intracellular cytokine staining demonstrated ChAdOx1 nCoV-19-mediated activation of memory CD4^+^ CD45RO^+^ T-cell populations

**Fig. 2:**
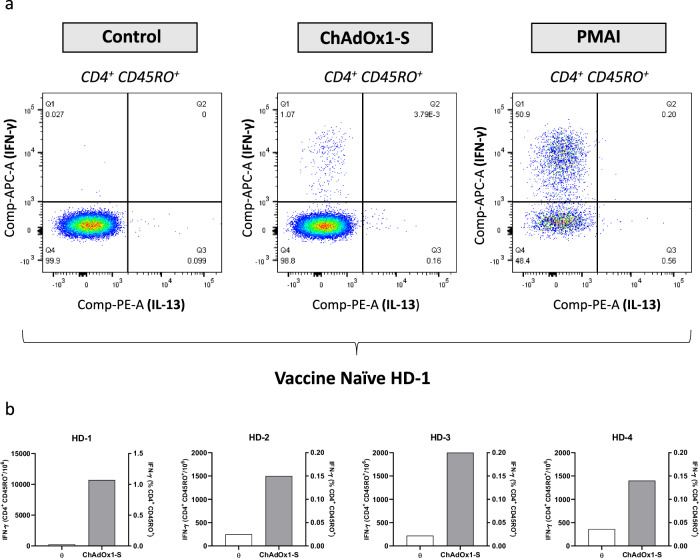
Intracellular cytokine staining (ICS) demonstrating IFN-γ secretion from circulating CD4^+^ CD45RO^+^ T-cells after stimulation with ChAdOx1 nCoV-19. **a** IFN-γ secretion from memory CD4^+^ CD45RO^+^ T-cell population after 18 h stimulation with ChAdOx1 nCoV-19 (100 vp/cell) in the presence of costimulatory antibodies and brefeldin A. Intracellular cytokine secretion is also demonstrated for PMAI (positive control). **b** IFN-γ secretion from memory CD4^+^ CD45RO^+^ T-cell populations (vaccine naïve HDs; *n* = 4). Data is displayed as IFN-γ producing cells per 1 × 10^6^ CD4^+^ CD45RO^+^ memory T-cells and as a percentage of stimulated T-cells within the gated CD4^+^ CD45RO^+^ population.

To identify specific T-cell populations activated by ChAdOx1 nCoV-19 within the conventional proliferative and cytokine-based T-cell assays performed, ICS analysis was conducted (Fig. 2). In the panel of 4 vaccine naïve HDs studied, CD4^+^ CD45RO^+^ memory T-cells were isolated as the primary activated population after stimulation with ChAdOx1 nCoV-19 (100 vp/cell) and assessment of IFN-γ secretion as marker of T-cell activation. In HD-1, >1% (1 × 10^4^ cells/10^6^) of memory CD4^+^ CD45RO^+^ cells were activated after ChAdOx1 nCoV-19 exposure, with PMAI successfully inducing widespread IFN-γ secretion as a positive control (Fig. 2a). This trend was consistent across all HDs studied, for which IFN-γ secretion was observed in 0.14–1% (1.4 × 10^4^–1 × 10^4^ cells/10^6^) of circulating CD4^+^ CD45RO^+^ memory T-cells (Fig. 2b).

### T-cells stimulated with ChAdOx1 nCoV-19 and Ad26.COV2. S secrete pro-inflammatory and cytotoxic cytokine mediators

**Fig. 3:**
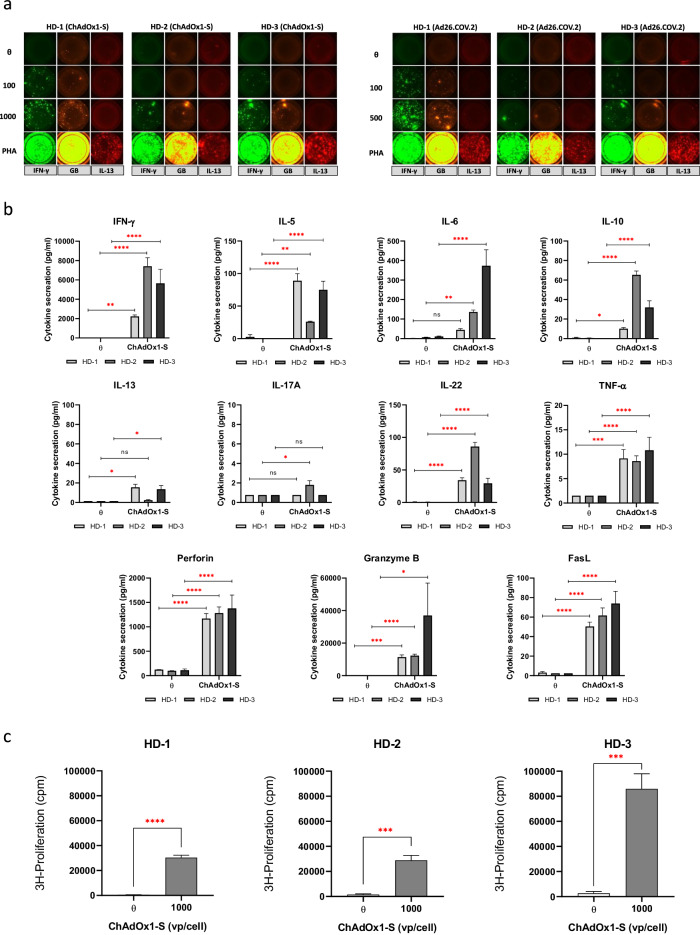
Cytokine profile displaying T-cell responses to ChAdOx1 nCoV-19 and Ad26.COV2. S in vaccine naïve donors. **a** T-cell cytokine profile (IFN-γ, granzyme B and IL-13) after 48 h exposure to ChAdOx1 nCoV-19 or Ad26.COV2. S at graded in vitro concentrations shown by Fluorospot analysis (*n* = 3). **b** Detailed cytokine profile following T-cell stimulation with ChAdOx1 nCoV-19 (1000 vp/cell) in vaccine naïve healthy donor PBMC (*n* = 3). Cytokine secretion was determined using the LEGENDplex BioLegend Custom Human 11-plex panel. Statistical significance was determined using a Student’s *t* or a one sample Wilcoxon test (*p < 0.01, **p < 0.001 ***p < 0.001, ****p < 0.0001). **c** Proliferative T-cell responses to ChAdOx1 nCoV-19 (1000 vp/cell) in vaccine naïve healthy donor PBMC (*n* = 3) used for LEGENDplex cytometric bead array immunoassays. Statistical significance was determined using a Student’s *t* (***p < 0.001, ****p < 0.0001). Data are presented as mean ± standard deviation of 3 experimental replicates.

Cytokine profiling was performed using PBMCs (2 × 10^5^ cells/well) isolated from 3 vaccine naïve HDs. Fluorospot Flex assays revealed the secretion of Th1 (IFN-γ) and cytotoxic effector molecules (granzyme B) after incubation with ChAdOx1 nCoV-19 and Ad26.COV2. S, with the release of Th2 (IL-13) cytokines notably absent (Fig. 3a). Additionally, cytokine release from PBMC taken from 3 vaccine naïve HDs was studied in greater depth using the LEGENDplex BioLegend Custom Human 11-plex cytokine panel after ChAdOx1 nCoV-19 (100 vp/cell) exposure (Fig. 3b). FACS analysis of the supernatant revealed the secretion of IFN-γ (>2000 pg/mL; *n* = 3), perforin (>1000 pg/mL; *n* = 3) and granzyme B (>10,000 pg/mL; *n* = 3) at high levels from stimulated T-cells. Additionally, the release of IL-5, IL-6, IL-10, IL-22, TNF-α and FasL was detected above background levels. Secretion of IL-13 and IL-17A was observed at reduced levels compared with other cytokines (2–20 pg/mL).

### Memory T-cell responses to ChAdOx1 nCoV-19 and purified ChAdOx1 are widespread in an extended cohort of pre-pandemic vaccine naive healthy donor PBMC

**Fig. 4:**
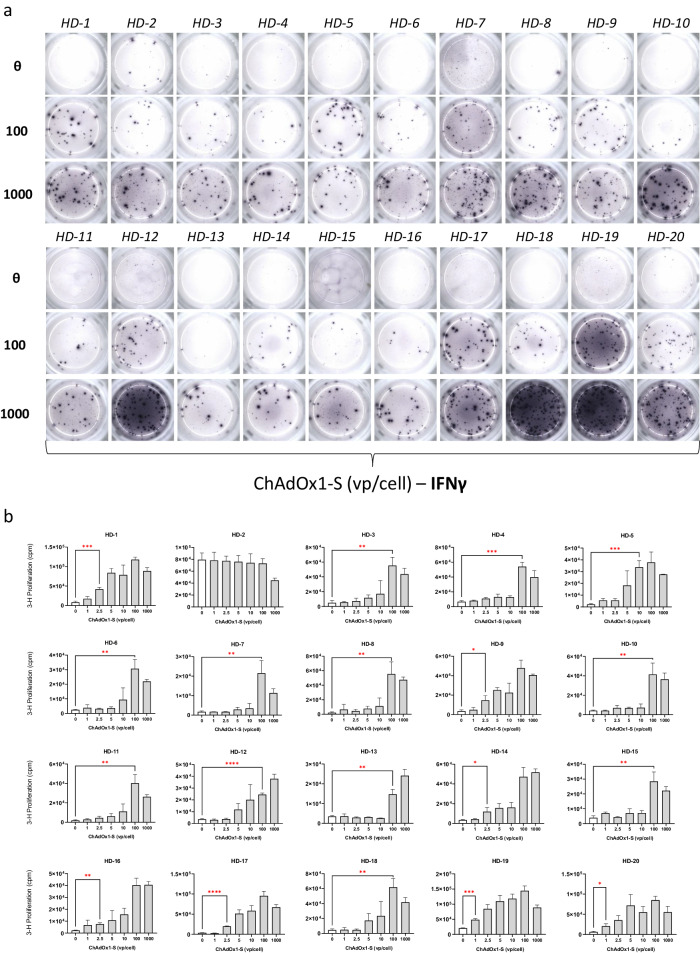
Proliferative and cytokine-based T-cell responses to ChAdOx1 nCoV-19 in healthy, vaccine naïve donors using extended PBMC cohort collected prior to the Covid-19 pandemic. **a** Cytokine release (IFN-γ) after 48 h exposure to ChAdOx1 nCoV-19 (0-1000 vp/cell) in pre-pandemic PBMC (*n* = 20) assessed using ELISpot assay. **b** Proliferative T-cell responses to ChAdOx1 (0–1000 vp/cell) in panel of pre-pandemic vaccine naïve HD PBMC (*n* = 20) naïve for ChAdOx1 nCoV-19 exposure. Statistical significance was determined using one-way ANOVA (*p < 0.01, **p < 0.001 ***p < 0.001, ****p < 0.0001). Data are presented as mean ± standard deviation of 3 experimental replicates.

**Fig. 5:**
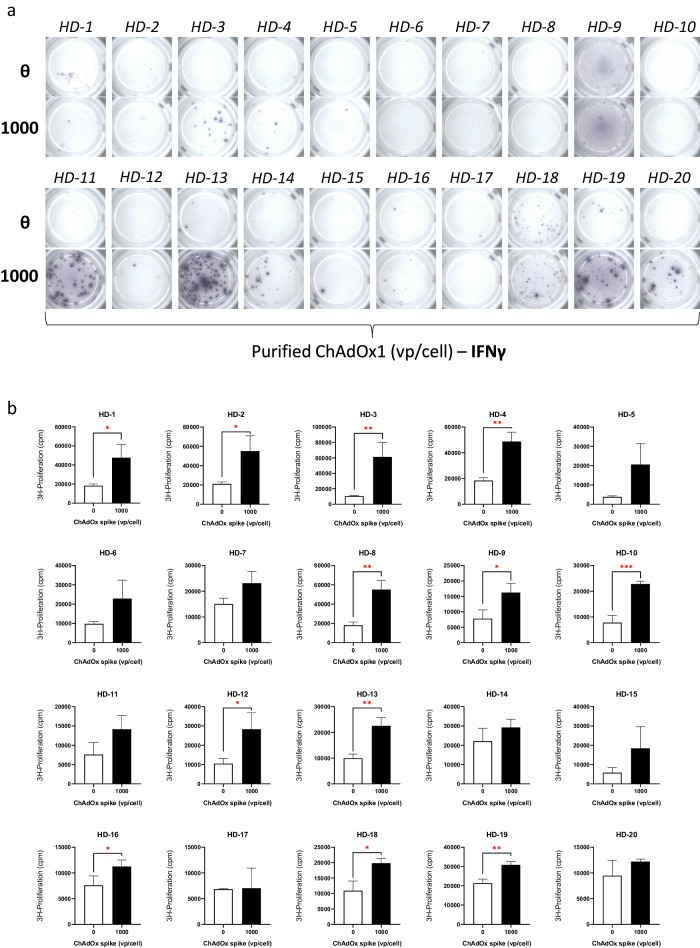
T-cell stimulation in vaccine naïve HDs after exposure to purified ChAdOx spike. **a** Cytokine release (IFN-γ) after 48 h exposure to ChAdOx spike (0–1000 vp/cell) in pre-pandemic PBMC assessed using ELISpot assay (*n* = 20). **b** Proliferative T-cell responses to ChAdOx spike (0–1000 vp/cell). Statistical significance was determined using one-way ANOVA (*p < 0.01, **p < 0.001 ***p < 0.001). Data are presented as mean ± standard deviation of 3 experimental replicates.

Due to T-cell stimulation observed after ChAdOx1 nCoV-19 exposure in 3 HDs, in combination with the low seroprevelence of ChAdY25 reported in the literature, further LTTs were conducted using an extended panel of 20 HDs (Fig. 4). The demographics of the healthy donor cohort used for this study can be viewed in Supplementary Table 1. IFN-γ was utilised as universal marker of immune stimulation. IFN-γ release was detected in all 20 HDs studied with strong responses present at both 100 and 1000 vp/cell of ChAdOx1 nCoV-19 (Fig. 4a). In the same HDs, proliferative T-cell responses were highly consistent, with 19/20 HDs displaying significant increases in lymphocyte proliferation (*P* < 0.05) (Fig. 4b). To exclude the activity of ChAdOx1 nCoV-19 excipients, T-cell responses were assessed after treatment with purified ChAdOx1 (1000 vp/cell) which possessed a genetically identical phenotype (Fig. 5). Analysis of a different panel of vaccine naïve HDs revealed cytokine release (IFN-γ) in <50% of selected donors after purified ChAdOx1 incubation (Fig. 5a). When studying proliferative T-cell responses 18/20 HDs demonstrated high levels of lymphocyte stimulation, with 12/20 responses reaching the threshold for statistical significance (Fig. 5b).

### ChAdOx1 nCoV-19 induces proliferative and cytokine-based T-cell stimulation in ChAdOx1 nCoV-19 vaccinated and Pfizer vaccinated controls

**Fig. 6:**
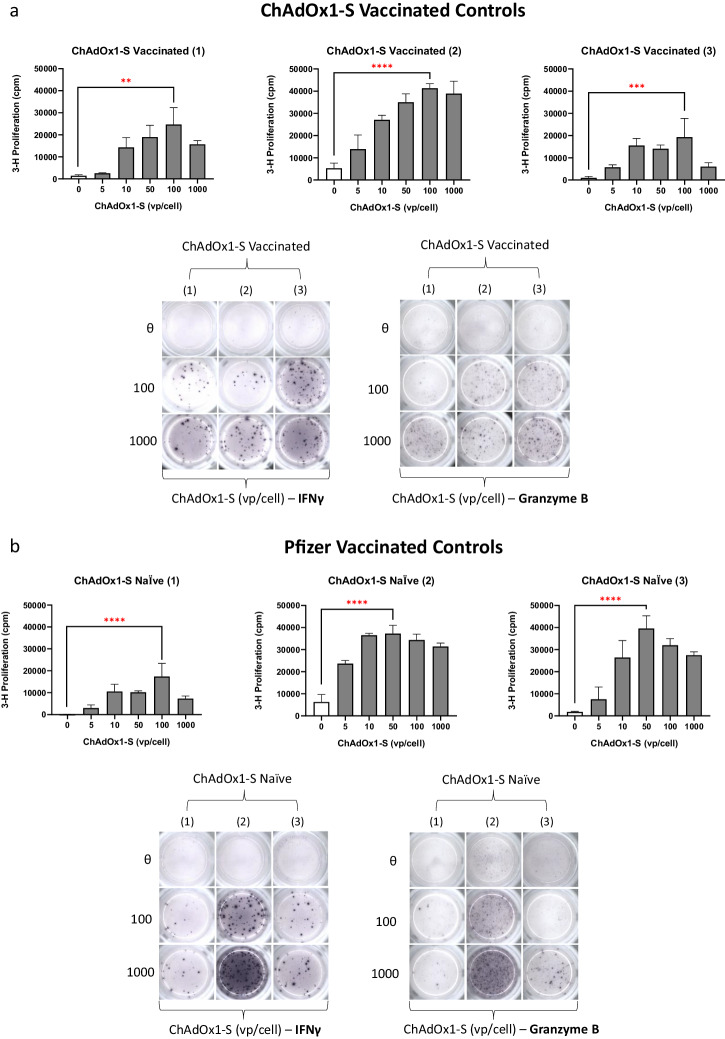
T-cell responses after ChAdOx1 nCoV-19 rechallenge in ChAdOx1 nCoV-19 vaccinated and Pfizer vaccinated (ChAdOx1 nCoV-19 naïve) controls. **a** T-cell proliferation and cytokine release (IFN-γ, granzyme B) showing immune stimulation of ChAdOx1 nCoV-19 (0-1000 vp/cell) in ChAdOx1 nCoV-19 vaccinated controls (*n* = 3). **b** T-cell activation (proliferation; IFN-γ, granzyme B) after ChAdOx1 nCoV-19 (0–100 vp/cell) exposure in Pfizer (BNT162b2) vaccinated controls (*n* = 3). Statistical significance was determined using one-way ANOVA (**p < 0.001 ***p < 0.001, ****p < 0.0001). Data are presented as mean ± standard deviation of 3 experimental replicates.

To delve deeper into the immunogenic nature of ChAdOx1, it was necessary to study T-cell responses in ChAdOx1 nCoV-19 vaccinated and Pfizer vaccinated controls (ChAdOx1 nCoV-19 naïve) for comparative analysis with data generated using pre-pandemic PBMC collected from vaccine naïve healthy donor models (Fig. 6). In PBMC samples collected from individuals vaccinated with ChAdOx1 nCoV-19 only (*n* = 3), lymphocyte responses were identified in all 3 donors after ChAdOx1 nCoV-19 incubation, with maximal T-cell proliferation (cpm > 1.8 × 10^4^) universally observed at 100 vp/cell (Fig. 6a). Cytokine release (IFN-γ and granzyme B) was studied in the same vaccinated individuals at optimal ChAdOx1 nCoV-19 concentrations (100–1000 vp/cell), with strong levels of Th1 and cytotoxic molecule secretion recorded (Fig. 6a). For individuals vaccinated with BNT162b2 and therefore naïve for ChAdOx1 nCoV-19 exposure, data was consistent with healthy donor models as proliferative (cpm > 1.6 × 10^4^) and cytokine-based (IFN-γ and granzyme B) T-cell activation was observed across all 3 Pfizer vaccinated controls at similar levels (Fig. 6b). Pfizer vaccinated controls were also assessed for memory T-cell stimulation following exposure to BNT162b2 (1–20 µg/mL) (Supplementary Fig. 2). Cytokine release assays (IFN-γ) demonstrated that BNT162b2 (20 µg/mL) was capable of inducing T-cell activation, with dose-dependent cytokine release observed within PBMC obtained from HC1 and HC2 (Supplementary Fig. 2a). No T-cell proliferation was observed after 5-day BNT162b2 exposure, with phytohemagglutinin (PHA) successfully demonstrating the proliferative capacity of T-cells within the assay (Supplementary Fig. 2b).

## Discussion

To date, studies investigating T-cell responses to SARS-CoV2 vaccines have almost exclusively focused on the activation of T-cells after SARS-CoV-2 Covid-19 vaccination and subsequent rechallange with SARS-CoV-2 spike proteins^5,7,8^. Here, we report that vaccines based on adenoviral vector delivery systems, specifically ChAdOx1 nCoV-19 and Ad26.COV2. S, directly activate T-cells from pre-pandemic vaccine naïve healthy donor PBMC. No memory T-cell responses were present after incubation with SARS-CoV-2 spike protein peptide pools (Fig. 1a, b) indicating no prior SARS-CoV-2 spike exposure. Proliferative and cytokine-based T-cell responses to ChAdOx1 were ubiquitous amongst the cohort of vaccine naïve HDs (Figs. 1, 3 and 4). This suggests the observed T-cell reactivity may be a result of cross-reactivity as humans are not frequently infected with ChAdY25 and therefore seroprevalence is low, although cross-species transmission is possible^29^. Differences in T-cell reactivity following exposure to Ad26.COV2. S were observed in HD-3 (Fig. 1), with cytokine release notably absent. This may be a result of the weak proliferative responses associated with HD-3 and inter-individual variability between healthy donor samples with differing levels of prior exposure.

Deletion of the E1 gene followed by the insertion of a transgene makes ChAdOx1 replication deficient and necessitates propagation in complementing cell lines, namely the HEK293 cell line^30^. Proteomic analysis performed by Greinacher et al., has now identified large numbers of virus production-derived HEK293 protein impurities present within ChAdOx1 nCoV-19. Interestingly, this study found that between 43% and 60% of the protein content of each ChAdOx1 nCoV-19 dose was attributed to proteins of HEK293 origin^31^, raising the question of the substantial, unknown, immunogenic potential of non-self-proteins incorporated within ChAdOx1.

ChAdOx1 nCoV-19 contains high levels of impurities, such as adenoviral and host-cell proteins, when compared to Ad26.COV2. S. It has been hypothesised that ChAdOx1 nCoV-19 contamination with adenovirus hexons and HEK293 cellular debris may be involved in VITT pathogenesis, arising from complex formation with platelets and the formation of polyanions that propagate an inflammatory environment^12,32^. Here, aberrant T-cell responses to excipients and impurities of ChAdOx1 nCoV-19 were ruled out after exposure to a genetically identical purified version of ChAdOx1, produced using a caesium chloride purification protocol (Fig. 5). It was also important to determine that the observed lymphocyte proliferation and cytokine activity following ChAdOx1 nCoV-19 exposure was of T-cell origin and not as a result of innate stimulation. Using CD3^-^ populations isolated from whole PBMC, lymphocyte activation was absent after incubation with ChAdOx1 nCoV-19 exposure (Supplementary Fig. 1). This demonstrates the importance of T-cells within response elicitation and excludes the possibility of activated NK cells masking proliferative and cytokine-based readouts.

The attachment of adenoviral vectors to cellular receptors, such as the T-cell receptor, is mediated by the adenovirus fibre knob protein^33^. Crystallography studies have indicated homology between the fibre knob protein associated with ChAdOx1 and other human adenoviruses, such as Ad5, despite sharing a relatively dissimilar amino acid sequence of 65%^34^. Candidate adenoviral vector platforms such as Ad26 and Ad35 have demonstrated genetic segregation from Ad5 with the aim of negating pre-existing immunity^35^. Additionally, several human adenoviruses are known to circulate at high levels, with Ad4 being the closest human relative to ChAdOx1 associated with cross-species transmission^36^. Therefore, it is possible that ChAdOx1 mediated T-cell activation may be caused by pre-existing cross-reactive immunity to specific T-cell epitopes present within circulating human adenoviruses. This is further reinforced by ICS studies that have demonstrated the activation of CD4^+^ CD45RO^+^ memory T-cell populations following short-term exposure (<24 h) to ChAdOx1 nCoV-19 within PBMC collected from vaccine naïve HDs pre-pandemic (Fig. 2). The presence of small fractions of reactive CD4^+^ CD45RO^+^ CD4+ T-cells is indicative of long-lived immunological memory to human adenoviruses with shared homology to ChAdOx1^37^.

Reactogenicity studies exploring the activation and phenotype of lymphocyte populations following ChAdOx1 nCoV-19 vaccination have reported activation of the adaptive immune system, confirmed by upregulation of activation (CD69) and proliferative markers (Ki-67^+^) in CD4+ and CD8+ T-cells^8^, with immune activation observed between 7 and 28 days post ChAdOx1 nCoV-19 vaccination. Our findings indicate lymphocyte responses after between 2-5 days of in vitro exposure in all groups assessed (pre-pandemic vaccine naïve HDs, ChAdOx1 nCoV-19 vaccinated controls and Pfizer vaccinated controls). This suggests the presence of pre-existing immunity as strong memory T-cell responses were detected a timepoints that exclude the possibility of a primed a T-cell response to ChAdOx1.

The Ad26.COV2. S vaccine was also associated with T-cell stimulation in HDs (Fig. 1a). As previously stated, the selection of Ad26 as a platform was due to the low seroprevalence associated with wild type Ad26 infection compared to other more commonly used serotypes such as Ad5, for which pre-existing immunity has long been a cause for concern^38^. Seroepidemiology studies have identified an Ad26 seropositive rate of between 50% and 70% in sub-Saharan African populations^24^. It should be noted that serological prevalence does not always equate to detectable T-cell reactivity as it is unlikely that every individual will be sensitised and possess antigen/peptide-specific T-cells circulating at the levels necessary for detection within T-cell assays. However, T-cell stimulation arising from Ad26.COV2. S exposure was perhaps surprising within the context of this study, with participants predominately of European origin. Proliferative T-cell responses were absent when studying responses in the same individuals to BNT162b2, as memory T-cell function would be negated within unexposed individuals and no possibility of viral cross-reactivity due to the nature of mRNA vaccines.

Exposure to both adenoviral vector vaccines, ChAdOx1 nCoV-19 and Ad26.COV2. S, was associated with the release of IFN-γ and granzyme B (Fig. 3a). T-cells from pre-pandemic vaccine naïve HDs were found to exhibit a cytokine profile aligned with a cytotoxic T-cell response, with the release of pro-inflammatory mediator’s indicative of early signalling for the amplification of both innate and adaptive immune responses to adenovirus vectors^39^. In the case of ChAdOx1 nCoV-19, these findings were further reinforced by flow cytometric studies analysing supernatant obtained from ChAdOx1 nCoV-19 stimulated PBMCs using LEGENDplex multi-panel cytokine immunoassays (Fig. 3b). Here, cytokine release was quantified and indicated that cytotoxic and cytolytic molecules, such as granzyme B, perforin and FasL, were secreted at high levels. The observed release of effector molecules from T-cells after ChAdOx1 nCoV-19 exposure aligns with known pathways of viral infection^40^, potentially mimicked in vitro by ChAdOx nCoV-19 within our studies. Adverse events, such as fever, swelling and localised pain, have been widely reported following ChAdOx1 nCoV-19 vaccination and recent studies have linked both local and systemic reactions with a vaccine-mediated inflammatory response^41,42^. In our study, the release of pro-inflammatory (IFN-γ and IL-6) and cytotoxic cytokine mediators from vaccine naïve HD PBMCs after ChAdOx1 nCoV-19 exposure may explain the reactogenicity associated with ChAdOx1.

It was important to compare T-cell responses to ChAdOx1 nCoV-19 between recently obtained vaccine naïve controls and vaccinated controls to further explore the widespread nature of lymphocyte activation and assess the validity of data produced from HD models (Fig. 6). Both proliferative and cytokine-based (IFN-γ and granzyme B) T-cell readouts were consistent between ChAdOx1 nCoV-19 vaccinated and Pfizer vaccinated controls, with no noticeable differences observed regarding the intensity and dosage at which T-cells are stimulated (Fig. 6a, b). These data are strongly aligned with ChAdOx1 nCoV-19-induced T-cell responses in pre-pandemic vaccine naive HD models and also concordant with prior ChAdOx1 exposure due to ability of immune cells to mount a strong memory T-cell response. These findings are most likely a result of cross-reactive T-cell responses that are pan-adenovirus in nature without direct specificity for ChAdOx1 (Y25). Studies assessing memory T-cell responses to Pfizer vaccinated (ChAdOx1 naïve) controls identified dose-dependent immune stimulation (IFN-γ release) after rechallange with BNT162b2 (Supplementary Fig. 2). This is consistent with prior BNT162b2 exposure which leads to long-lasting immunological memory in the form of a robust T-cell response^43,44^.

Previous studies have defined immunodominant T-cell epitopes on both the hexon and penton proteins derived from human adenoviruses, restricted by HLA class I (HLA-A*02) and II (HLA-DP4) alleles of high prevalence^45,46^. Here, we speculate that T-cell reactivity to adenoviral-based COVID-19 vaccines within our cohort of unexposed healthy donors are most likely a result of widespread recognition of immunodominant T-cell epitopes present at high levels within the population. A similar phenomenon has been reported when studying IgG antibody responses to conserved SARS-CoV-2 spike subdomains^47^. In this study, SARS-CoV-2 was not recognised by the adaptive immune system as a novel pathogen and cross-reactivity with shared immunodominant regions between seasonal betacoronaviruses correlated with COVID-19 disease severity.

In summary, our findings demonstrate the omnipresent nature of strong, cross-reactive CD4^+^ CD45RO^+^ memory T-cell responses to ChAdOx1 and Ad26 in healthy, vaccine naïve PBMC samples collected prior to the Covid-19 pandemic. Although previous exposure to chimpanzee adenoviruses has been reported in certain demographics, seroprevalence is low and inconsistent with the level of responses observed in vaccine naïve donors. Consequently, this suggests that immune stimulation after ChAdOx1 nCoV-19 exposure may arise from pre-existing immunity to prevalent human adenoviruses with shared homology between T-cell epitopes. Moving forward, it will be necessary to define specific epitope(s) involved to potentially inform on vaccine development. This may be achieved using a proteomic-based approach to elute and synthesise MHC-bound peptides spanning the major structural proteins of adenoviral vectors.

## Methods

### Sample collection and ethical approval

Vaccine naïve PBMCs were accessed through the Liverpool healthy donor biobank which contained 1200+ cryopreserved and HLA typed PBMC samples collected prior to the Covid-19 pandemic. Fresh venous blood samples (40 mL) were taken from vaccinated controls using lithium heparin coated tubes. Written, informed consent was given by all healthy volunteers used in this study and ethical approval was obtained from the Liverpool research ethics committee.

### Isolation of peripheral blood mononuclear cells

Whole venous blood was collected using vacutainer heparinised tubes from ChAdOx1 nCoV-19 vaccinated and Pfizer vaccinated controls. Peripheral blood mononuclear cells (PBMCs) were isolated by density gradient centrifugation, during which whole blood was layered onto an equal volume of lymphoprep™ (STEMCELL^™^ Technologies) and then centrifuged for 25 min at 2000 rpm with no brake applied. The resulting buffy coat, comprising the lymphocyte layer, was carefully removed by gentle resuspension using a Pasteur pipette. Cells were washed twice with HBBS buffer solution before resuspension in RPMI-based T-cell culture media supplemented with penicillin (100 μg/mL), streptomycin (100 U/mL), holo-transferrin (12.5 mg/mL), HEPES buffer (25 mM), L-glutamine (2 mM) and 10% human AB serum. The cell suspension was stained with trypan blue (0.2% w/v) and counted using a Neubauer haemocytometer. The overall cell viability was determined using trypan blue exclusion, in which uptake of trypan by cells indicated a permeabilized membrane and was indicative of cell death, with viabilities >95% typically achieved. CD3^-^ populations were isolated from whole PBMC populations using magnetic bead separation. This was achieved by negative selection of CD3^+^ cells using a Pan-T antibody cocktail and performed according to the manufacturer’s instructions (Miltenyi Biotec, UK).

### ChAdOx1 purification

Purified ChAdOx1 was generated using a double caesium chloride gradient virus purification protocol according to methodologies previously described^34^. HEK-293 T-Rex cells grown in DMEM media (Sigma Aldrich) supplemented with 10% FCS (Sigma Aldrich), 1% Penicillin/Streptomycin (Gibco) (complete media) were used to grow the ChAdOx1 vector. For infection, media was removed from ten 150 cm^2^ flasks of HEK-293 T-Rex cells at 60–70% confluence were washed with PBS, and the media replaced with 15 ml of complete media containing ChAdOx1 at 0.1–1 vp/cell. Cells were observed until a noticeable cytopathic effect (CPE) was evident, at which point the cells were pooled and were collected by centrifugation (1,5000 rpm; 5 min). Media was discarded and the cell pellet was resuspended in 5 mL phosphate-buffered saline (PBS) before thorough mixing with tetrachloroethylene (1:1). The resultant mixture was centrifuged (2000 rpm; 20 min) followed by removal of the top layer of aqueous solution. The sample was further purified by additional centrifugation and removal of the solutions top aqueous layer, with the remaining steps of the purification protocol performed according to the two-step caesium chloride gradient methodology described by Uusi-Kerttula et al.^48^. Virus was titered using a micro BCA assay against a range of protein standards with viral titer deduced assuming that 1 μg protein = 4 × 10^9^ viral particles. Viral purity and homogeneity were additionally confirmed using a Nanosight NS300 (Malvern Panalytical).

### Lymphocyte transformation test

PBMCs from vaccine naïve healthy volunteers and vaccinated controls were isolated from fresh venous blood by density gradient centrifugation as previously described. For the assessment of T-cell responses to SARS-CoV-2 COVID-19 vaccines using samples collected pre-pandemic, PBMCs were previously cryopreserved in a solution containing 80% FBS and 20% DMSO. Cryopreserved PBMCs were thawed, washed in T-cell culture medium and incubated for 6 h prior to assay commencement. PBMC were diluted in T-cell culture medium to achieve a seeding density of 1.5 × 10^5^ cells/well and transferred into a U-bottomed 96 well-plate in triplicate cultures. PBMC were incubated with SARS-CoV-2 COVID-19 vaccines at graded concentrations for a period of 5 days (37 °C, 5% CO2). The inclusion of conditions in which PBMC were exposed to phytohemagglutinin (10 μg/mL) represented a positive control for the assay by way of detecting non-specific lymphocyte proliferation. On day 5, [3H]-thymidine was added to each assay condition at a concentration of 0.5 μCi/well, and incubated for a further 16 h. On day 6, plates were harvested onto printed fibreglass filter mats using a cell harvester (Harvester 96 Tomtec; Tomtec Imaging Systems GMbH, Unterschleissheim, Germany) and dried at 80 °C. Fibreglass mats were heat sealed inside Wallac sample bags with MeltiLex scintillator sheets using a Wallac 1495-021 Microsealer (Perkin Elmer, Waltham, MA, USA). Radioactivity incorporated within the DNA of proliferating cells was detected and quantified using a MicroBeta 2450 microplate counter and readouts were given by counts per minute (cpm).

### Enzyme-linked immunospot assay

Cytokine release from PBMCs after SARS-CoV-2 COVID-19 vaccine exposure was performed by enzyme-linked immunospot (ELISpot) assay. Protein Binding Immobilon-P Membrane 96-well Multiscreen® filter plates were activated by the addition of 35% ethanol solution before thorough washing (5x) with 200 μL distilled water. Activated Multiscreen® filter plates were then coated with 100 μL of IFN-y (Cat. No 3420-3, 1:100 dilution) or granzyme B (Cat. No 3486-1, 1:50 dilution) capture antibody diluted in sterile HBSS according to the manufacturer’s instructions (Mabtech, Nacka Strand, Sweden). Coated plates were then incubated for 24 h at 4 °C. On the day of the assay PBMCs were washed in T-cell culture medium to exclude any cytokine milieu and seeded at 2 × 10^5^ cells/well. PBMCs were incubated in pre-coated Multiscreen® filter plates with SARS-CoV-2 COVID-19 vaccines at graded concentrations for 48 h. For assay development, cells were discarded and ELISpot plates were washed with 200 μL HBSS. Detection antibodies were conjugated with biotin corresponding to the cytokines of interest and diluted in HBSS supplemented with 0.5% FBS according to the manufacturer’s instructions. Biotinylated antibody solution was added to each well and incubated for 2 h. Following incubation, plates were washed with HBSS and streptavidin conjugated alkaline phosphatase (1:1000 dilution) was added to each well of the assay and plates were incubated for 1 h. Sterile filtered (0.45 μM) BCIP-NBT substrate solution was added to the assay and plates were incubated at RT for 15–20 min in the dark to allow colorimetric reaction to occur. Spots were imaged using an AID ELISpot reader (Cadima Madical, Stourbridge, UK).

### Intracellular cytokine staining

Intracellular cytokine staining (ICS) assays were performed according to methods described by Peng et al., using PBMC from vaccine naïve HDs collected pre-pandemic^8^. Briefly, PBMCs (1 × 10^6^) were stimulated with either cell culture medium, ChAdOx1 nCoV-19 (100 vp/cell) or PMAI, in the presence of costimulatory anti-CD28 and anti-CD49 (BD Biosciences; 1 µg/mL) for 2 h (37 °C, 5% CO2). After incubation, brefeldin A (10 µg/mL) was added and cells were incubated for a further 18 h. Samples were then stained to isolate specific T-cell populations including CD3 (Cat. No. 300448, 1:50 dilution), CD4 (Cat. No. 555346, 1:30 dilution), CD8 (Cat. No. 301036, 1:100 dilution) and CD45RO (Cat. No. 304222, 1:50 dilution) populations (BioLegend®). Intracellular cytokine secretion from individual immune populations was identified using IFN-γ (Cat. No. 17-7619-82, 1:200 dilution) and IL-13 (Cat. No. 12-7136-42, 1:25 dilution) fluorophore-conjugated antibodies (eBioScience). Fluorescent signals were measured using BD FACSCanto II and analysed using FlowJo version 10 software. The gating strategy used can be viewed in Supplementary Fig. 3.

### Fluorospot Flex assay

To study the release of multiple cytokines from a single PBMC population after 48 h SARS-CoV-2 COVID-19 vaccine exposure, Fluorospot Flex assays, customised for the simultaneous detection of IFN-γ, IL-13 and granzyme B, were performed. Capture and biotinylated antibodies were amalgamated for the cytokines of interest and diluted using 0.1% BSA. Otherwise, Fluorospot Flex assays were performed according to the manufacturer’s instructions and in line with methodologies previously described for the ELISpot assay. Fluorescent Spots were imaged using an AID *i*Spot Fluorospot reader (Cadima Madical, Stourbridge, UK).

### Cytometric bead array immunoassay

Supernatant from vaccine naïve HD PBMC (*n* = 3) were incubated (37 °C, 5% CO2) for 5 days with ChAdOx1 nCoV-19 (100 vp/cell), according to the experimental procedure previously described for the LTT. Harvested supernatant (25 μL) was used for cytokine quantification using a bead-based immunoassay according to the manufacturer’s instructions (LEGENDplex, BioLegend Custom Human 11-plex panel). Specific antibody coated beads were incubated with supernatant pooled from triplicate wells for each condition forming an analyte-antibody complex. After washing, a biotinylated detection antibody cocktail was added which bound to the specific analyte-antibody complexes. Streptavidin-phycoerythrin was subsequently added which bound to the biotinylated detection antibodies, providing fluorescent signal intensities in proportion to the bound analyte amount. Fluorescent signals were measured using BD FACSCanto II and analysed using LEGENDplex data analysis software where concentrations of each analyte were determined using a standard curve generated from the same assay.

### Statistical analysis

Statistical analysis was performed using GraphPad Prism 9.0 software and data are presented as mean ± standard deviation of 3 experimental replicates. To evaluate proliferative T-cell responses for which data was found to be normally distributed, one-way ANOVA and students t-tests were performed. For data containing identical values that are not due to chance, a one sample Wilcoxon test was performed. For these tests, *p < 0.05 was deemed to be the threshold for statistical significance.

### Reporting summary

Further information on research design is available in the Nature Research Reporting Summary linked to this article.

### Supplementary information

Supplementary Material

Reporting Summary

## Data Availability

The data that support the findings of this study are available from the corresponding author upon reasonable request.
