# Supplementary material for: Identification of cross reactive T cell responses in adenovirus based COVID 19 vaccines
Source: NPJ Vaccines. 2024 Jun 5;9:99. doi: 10.1038/s41541-024-00895-z (PMC11153626; doi:10.1038/s41541-024-00895-z)
Supplement: Supplementary file 1 — Supplementary Material [file 41541_2024_895_MOESM1_ESM.pdf]

CD3⁻ (Non T-cell Population)

a

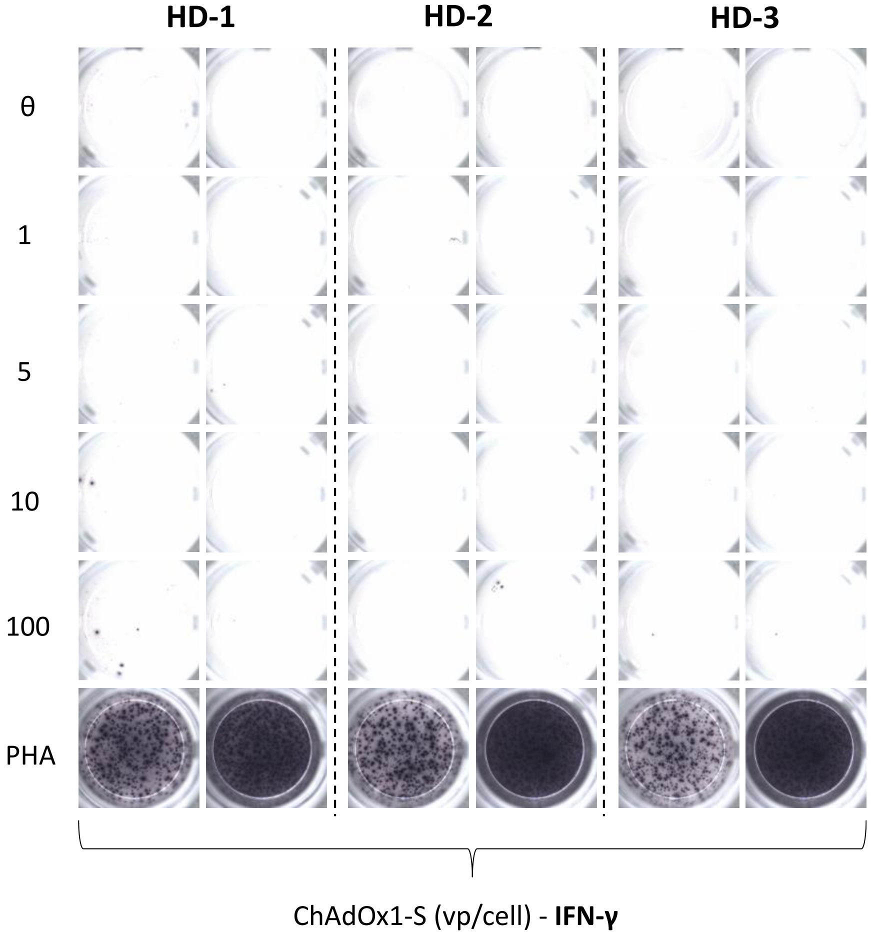

b

CD3⁻ (Non T-cell Population)

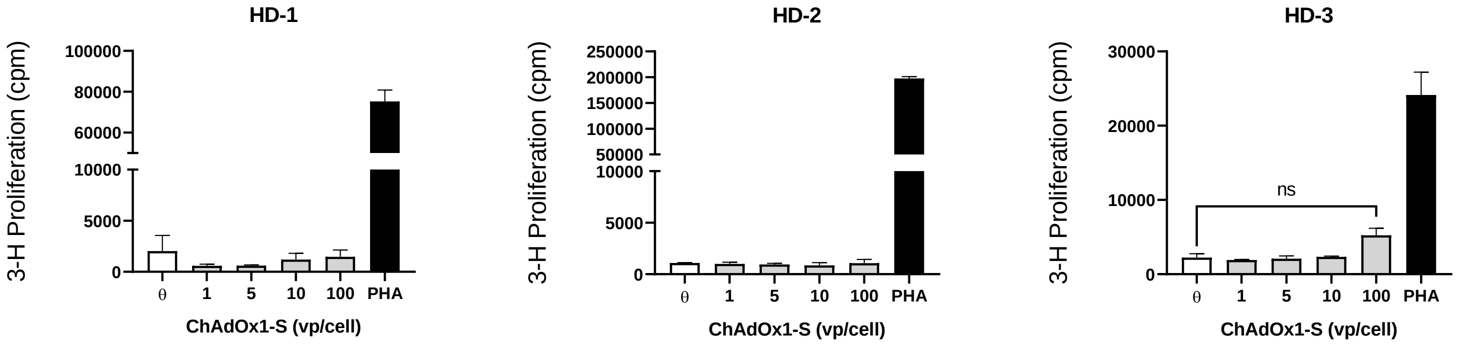

Supplementary Figure 1: Proliferation and cytokine release from $CD3^-$ populations isolated from healthy, vaccine naïve donors after ChAdOx1 nCov-19 incubation. (a) Non-CD3 cytokine release (IFN-γ) determined by ELISpot after exposure to ChAdOx1 nCov-19 (0 – 100 vp/cell) in healthy, vaccine naïve donors (n=3). (b) Non-CD3 proliferation determined by 3-H incorporation T after ChAdOx1 nCov-19 (0 – 100 vp/cell) exposure in the same panel of healthy donors (n=3). Phytohemagglutinin (PHA) was used as a positive control within the assay to demonstrate the proliferative capacity of T-cells. Data are presented as mean ± standard deviation of 3 experimental replicates.

Pfizer Vaccinated Controls

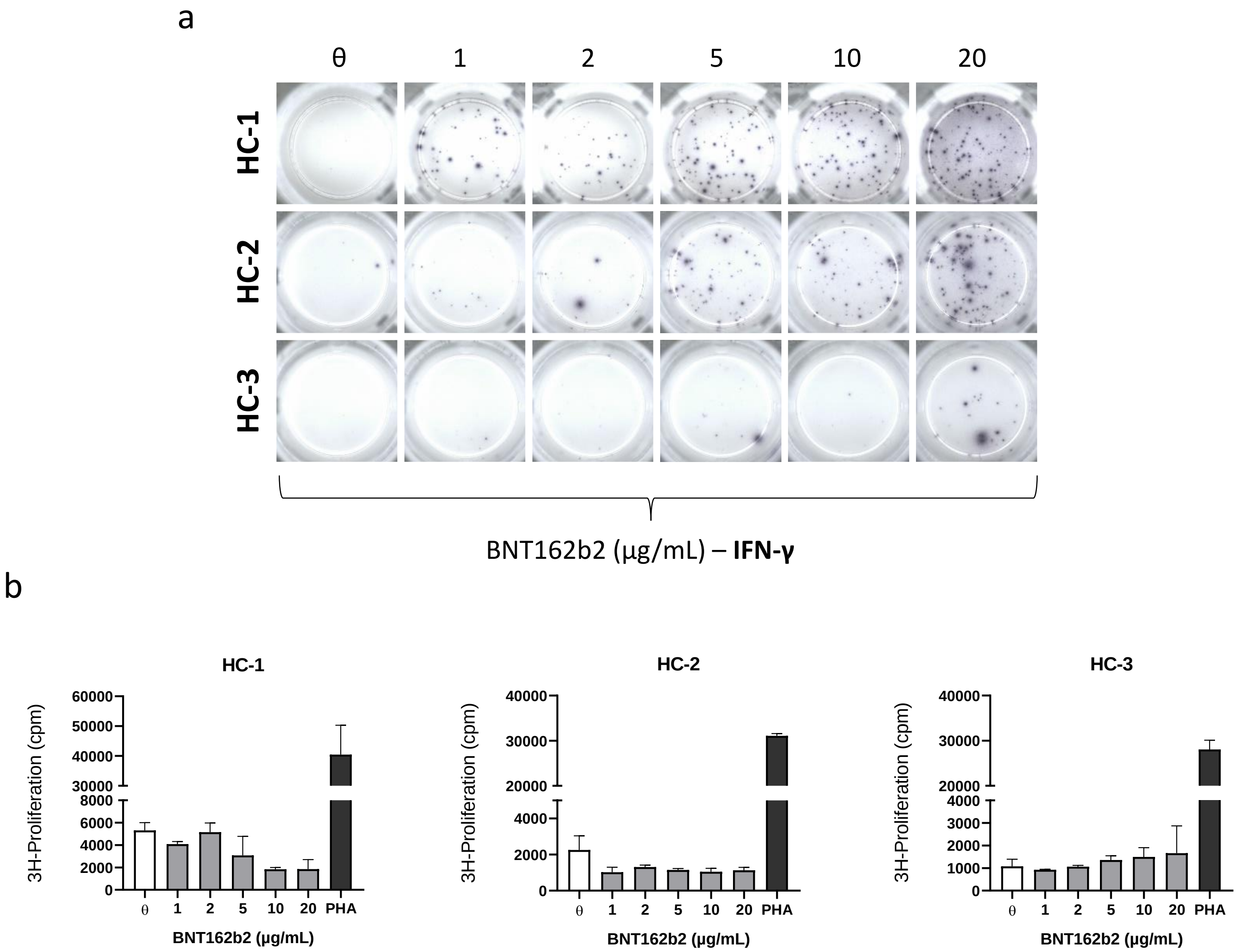

Supplementary Figure 2: T-cell responses after Pfizer (BNT162b2) rechallenge in Pfizer vaccinated healthy controls, determined by lymphocyte transformation test. (a) T-cell cytokine release (IFN-γ) showing BNT162b2-induced immune stimulation in Pfizer (BNT162b2) vaccinated healthy controls after exposure to graded concentrations of BNT162b2 (0 – 20 µg/mL). (b) T-cell proliferation after BNT162b2 exposure (0 – 20 µg/mL) in vaccinated healthy controls (n=3). Phytohemagglutinin (PHA) was used as a positive control within the assay to demonstrate the proliferative capacity of T-cells. Data are presented as mean ± standard deviation of 3 experimental replicates.

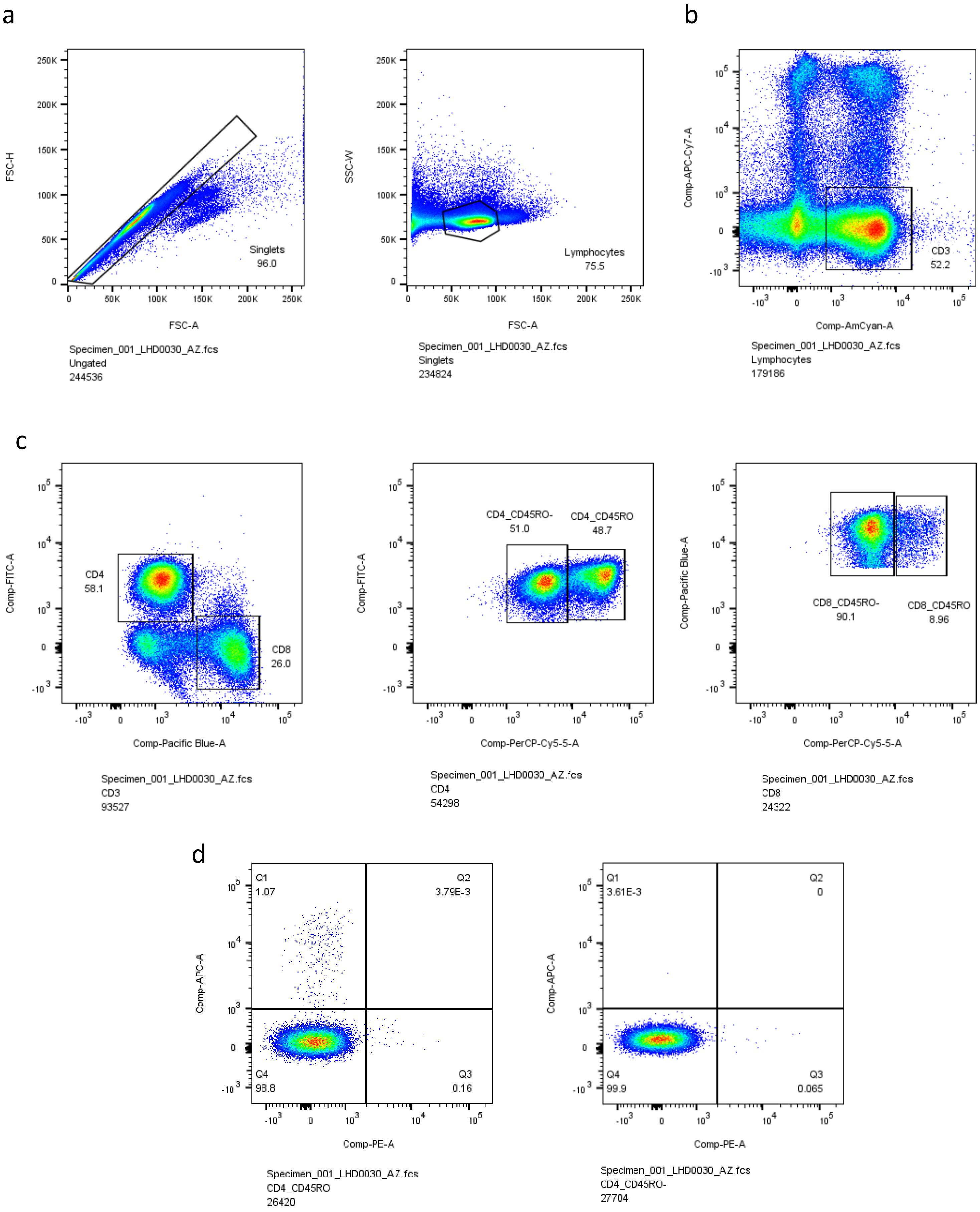

Supplementary Figure 3: Gating strategy for ICS analysis of ChAdox1-induced T-cell responses. (a) PBMCs were gated based on FSC-A and SSC-A parameters. (b) Gating of CD3 lymphocyte population using AmCyan-A. (c) Gating of CD4+ and CD8+ T-cell population using FITC-A and Pacific Blue-A. Each T-cell population was further gated for CD45RO+ (memory) and CD45RO- (naïve) populations. (d) IFNγ (APC-A) was detected in the CD4+ CD45RO+ population.

Supplementary Table 1: Demographics of healthy donors used in T-cell assays for the study of T-cell responses to adenovirus-based COVID-19 vaccines.

Donor ID	Sex	Age	Year sample obtained
HD-1	F	46	2013
HD-2	M	46	2013
HD-3	M	45	2013
HD-4	M	35	2013
HD-5	F	30	2013
HD-6	M	27	2013
HD-7	F	45	2013
HD-8	M	45	2013
HD-9	M	18	2013
HD-10	M	46	2013
HD-11	M	37	2013
HD-12	F	39	2013
HD-13	M	25	2013
HD-14	M	24	2013
HD-15	F	52	2013
HD-16	F	22	2013
HD-17	M	22	2013
HD-18	M	36	2013
HD-19	F	28	2013
HD-20	F	56	2013

Supplementary Figure Legends

Supplementary Figure 1: Proliferation and cytokine release from CD3$^-$ populations isolated from healthy, vaccine naïve donors after ChAdOx1 nCov-19 incubation. (a) Non-CD3 cytokine release (IFN-γ) determined by ELISpot after exposure to ChAdOx1 nCov-19 (0 – 100 vp/cell) in healthy, vaccine naïve donors (n=3). Data are presented as mean ± standard deviation of 3 experimental replicates. (b) Non-CD3 proliferation determined by 3-H incorporation T after ChAdOx1 nCov-19 (0 – 100 vp/cell) exposure in the same panel of healthy donors (n=3). Phytohemagglutinin (PHA) was used as a positive control within the assay to demonstrate the proliferative capacity of T-cells.

Supplementary Figure 2: T-cell responses after Pfizer (BNT162b2) rechallenge in Pfizer vaccinated healthy controls, determined by lymphocyte transformation test. (a) T-cell cytokine release (IFN-γ) showing BNT162b2-induced immune stimulation in Pfizer (BNT162b2) vaccinated healthy controls after exposure to graded concentrations of BNT162b2 (0 – 20 µg/mL). (b) T-cell proliferation after BNT162b2 exposure (0 – 20 µg/mL) in vaccinated healthy controls (n=3). Phytohemagglutinin (PHA) was used as a positive control within the assay to demonstrate the proliferative capacity of T-cells. Data are presented as mean ± standard deviation of 3 experimental replicates.

Supplementary Figure 3: Gating strategy for ICS analysis of ChAdOx1-induced T-cell responses. (a) PBMCs were gated based on FSC-A and SSC-A parameters. (b) Gating of CD3 lymphocyte population using AmCyan-A. (c) Gating of CD4+ and CD8+ T-cell population using FITC-A and Pacific Blue-A. Each T-cell population was further gated for CD45RO+ (memory) and CD45RO- (naïve) populations. (d) IFNγ (APC-A) was detected in the CD4+ CD45RO+ population.

Supplementary Table 1: Demographics of healthy donors used in T-cell assays for the study of T-cell responses to adenovirus-based COVID-19 vaccines.
